# Comparing Proteomics and RISC Immunoprecipitations to Identify Targets of Epstein-Barr Viral miRNAs

**DOI:** 10.1371/journal.pone.0047409

**Published:** 2012-10-16

**Authors:** Malika Kuzembayeva, Ya-Fang Chiu, Bill Sugden

**Affiliations:** McArdle Laboratory for Cancer Research, University of Wisconsin, Madison, Wisconsin, United States of America; Louisiana State University Health Sciences Center, United States of America

## Abstract

Epstein-Barr virus is a gamma-herpes virus that is causally associated with several lymphomas and carcinomas. This virus encodes at least 25 pre-miRNAs, which are expressed in infected cells to yield more than 50 detected mature miRNAs. miRNAs are small, non-coding RNAs that inhibit gene expression by promoting the inhibition of translation or of degradation of mRNAs. Currently, the function of these viral miRNAs and the contribution they provide to EBV’s life-cycle remain largely unknown, due to difficulties in identifying cellular and viral genes regulated by these miRNAs. We have compared and contrasted two methods to identify targets of viral miRNAs in order to identify the advantages and limitations of each method to aid in uncovering the functions of EBV’s miRNAs.

## Introduction

miRNAs are small, noncoding RNAs of about 20–22 nucleotides in length that regulate gene expression at the post-transcriptional level. They have been implicated as regulators of most cellular processes with abnormal expression of miRNAs reported in various diseases including cancer [1], [2]. Regulation of gene expression by the miRNAs involves their binding to complimentary sequences in target mRNAs, and guiding the RNA Induced Silencing Complexes (RISCs) to those mRNAs, resulting in a combination of mRNA degradation and inhibition of translation. The biogenesis of miRNAs and the modes of their regulation have been extensively reviewed [3], [4].

Epstein-Barr virus (EBV) is a gamma herpes virus that infects more than 90% of the world’s adult population and was the first virus identified to encode many miRNAs. Its miRNAs are encoded in two transcripts, the BamHI H Rightward Fragment 1 (BHRF1) transcript and the BamHI A Rightward transcript (BART) [5], [6]. While EBV usually persists as an asymptomatic, lifelong infection, it is causally associated with several malignancies including Burkitt’s and Hodgkin’s lymphomas, gastric and nasopharyngeal carcinomas (NPC), and post-transplant lymphoproliferative disease [7], [8]. The latent phase of EBV’s life-cycle, a phase in which no viral progeny are produced, is also characterized by the expression of different viral genes in the infected cell, but always include the miRNAs encoded by the BART transcript [6], [9].

Currently few mRNAs potentially regulated by EBV’s miRNAs have been identified, and the biological significance of this miRNA-mediated regulation is poorly understood. Among viral targets of the miRNAs, miR-BART2 was reported to decrease the expression of the viral DNA polymerase BALF5, suggesting that miR-BART2 may inhibit the transition from latent to productive viral replication [5], [10]. EBVs Latent Membrane Protein 1 (LMP1) was reported to be regulated by several BART miRNAs *in vitro* in NPC and Burkitt’s lymphomas [11], [12]. As for cellular targets, miR-BART5 was reported to regulate the pro-apoptotic gene, p53 upregulated modulator of apoptosis (PUMA), in epithelial cells, which was thought to promote host survival [13]. Viral miRNAs of the BHRF1 locus have also been shown to inhibit apoptosis and promote proliferation of primary B cells shortly following infection with EBV [14]. Recently, J. Haas et al have also confirmed two genes involved in cellular transport to be regulated by EBV’s miRNAs [15], and B. Cullen and colleagues have provided a comprehensive survey of potential targets of these miRNAs in lymphoblastoid cells implicated in a variety of cellular processes [16].

One major impediment to elucidating functions of viral miRNAs is their subtle effects on gene expression and the complex nature of interactions among miRNAs and their target transcripts [17], [18]. Multiple studies have shown that miRNAs have small effects on individual targets, rendering traditional methods employed to elucidate these interactions insufficiently sensitive, and making it difficult to validate authentic targets. miRNAs of EBV appear to be expressed at much lower levels than cellular miRNAs which only further complicates the identification of transcripts regulated by these miRNAs [9]. A second barrier to finding the roles of viral miRNAs in the viral life-cycle comes from viral miRNAs not being conserved as are both cellular miRNAs and their targeted mRNAs [19]. This lack of conservation precludes one major strength of bioinformatical approaches used to identify targets of cellular miRNAs.

While the human genome is approximately 10,000 times larger than that of EBV, it encodes only 50 times as many miRNAs. The enrichment for miRNAs by EBV makes the viral miRNAs fascinating to study. Our understanding of the functions and the advantages provided by the viral miRNAs however remains limited due to the lack of functional studies and identified bona fide targets of EBV miRNA-mediated regulation. Identifying cellular gens inhibited by the viral miRNAs will help elucidate pathways regulated by these miRNAs. We have therefore used two approaches, 2D gel separation coupled with mass spectrometry, and RISC immunoprecipitation (RISC IP) coupled with deep sequencing to compare approaches, to understand their advantages and limitations and to identify the method of choice to aid our understanding of the functional importance of viral miRNAs in the life cycle of EBV. The approach using 2D gel separation has the advantage of detecting changes in levels of a protein directly and has been used successfully to identify mRNAs regulated by miRNAs [20], [21], while that using RISC IPs has the advantage of surveying a greater proportion of the mRNAs that can be regulated by miRNAs.

## Materials and Methods

### Cell Lines, Culture Methods and RNA Isolation

EBV-negative Burkitt’s lymphoma cell line, BJAB (provided by Dr. Elliott Kieff [22]), was cultured in RPMI 1640 (Invitrogen) supplemented with L-glutamine, 10% fetal bovine serum (FBS), and antibiotics (200 U/mL penicillin and 200 µg/mL streptomycin). All cells were incubated in 5% CO_2_ at 37°C. The viability of cell lines was assessed by Trypan Blue staining and by evaluating overall cell morphology. Only cultures with a viability of more than 95% were used in this study. BJAB BARTs cells express EBV’s BART miRNAs constitutively via a retroviral vector, where BJAB Empty cells were transduced with an empty retroviral vector as described previously (D. Vereide et al, manuscript submitted). Total RNA was isolated using TRIzol reagent (Invitrogen), and precipitated with linear acrylamide (Ambion) as a carrier as previously described [9].

**Figure 1:**
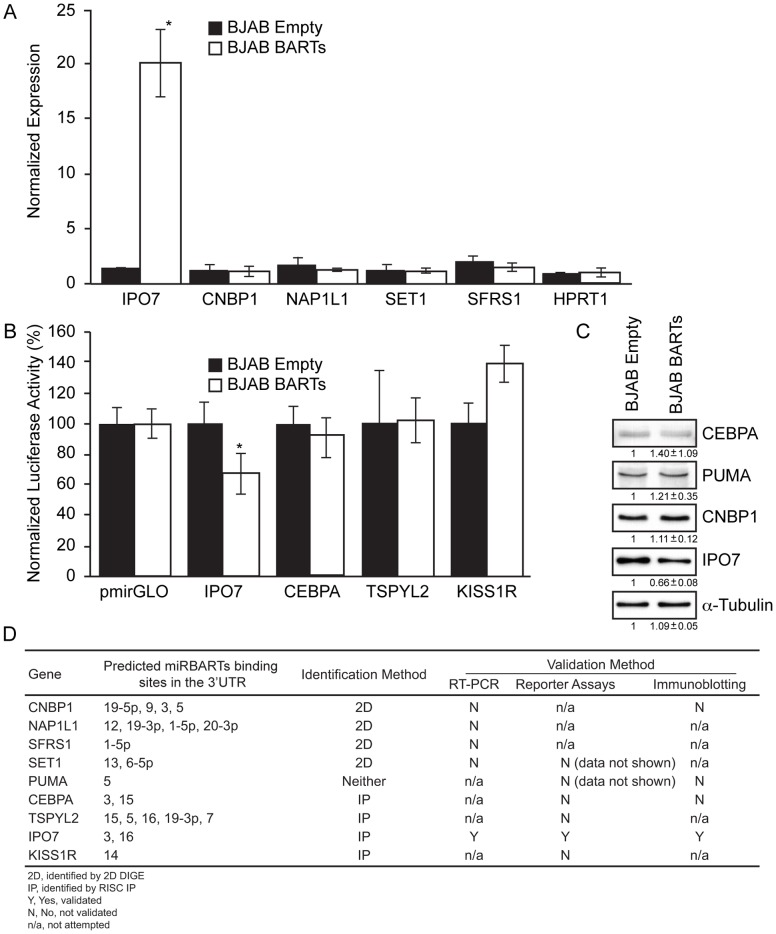
Validating the candidate genes obtained via 2D DIGE and RISC IPs. A. Measuring levels of candidate transcripts obtained by RISC IPs in Ago2-immunoprecipitated RNA samples of BJAB BARTs and BJAB Empty cell lines by RT-PCR revealed an enrichment for IPO7 transcripts in immunoprecipitates of BJAB BARTs cells (*indicates p-value <0.0005, Student’s t-test). B. Reporter constructs containing the 3′UTRs of candidate genes were transfected into BJAB BARTs and BJAB Empty cells. The normalized luciferase activity in BJAB Empty cells was arbitrarily set to 100% (*indicates p-value <0.05, Student’s t-test). The luciferase activity of IPO7 3′UTR-pmiRGLO construct in BJAB BARTs cells decreased by 33%. C. Expression levels of CEBPA, PUMA, CNBP1 and IPO7 in BJAB Empty and BJAB BARTs cells were analyzed by immunoblotting, quantified by Image J software and normalized to levels of alpha-tubulin. The levels of expression of all proteins are indicated under the respective bands. The levels of IPO7 protein were found to decrease by 33% in the presence of BART miRNAs in three biological replicates while the levels of the other three proteins were unaffected by them. D. Nine candidates identified either by 2D DIGE or RISC IPs were examined further either by RT-PCR to measure their levels in RISCs, in reporter assays, or by immunoblotting where appropriate antibodies were available. IPO7 was validated by all three methods.

**Table 1:** Measuring the effects of BART miRNAs on expression levels of cellular proteins using 2D DIGE.

Spot	Protein	NCBI AccessionNumber	Gene	Fold change of protein (BJAB BARTs/BJAB Empty)
1	Heat shock 70 kDa protein 5	GI: 16507237	HSPA5	−1.31
2	Heat shock 90 kDa protein 1, beta	GI: 20149594	HSP90AB1	−1.45
3	Heat shock 70 kDa protein 9 precursor	GI: 24234688	HSPA9	−1.38
5;6	Gelsolin isoform b	GI: 38044288	GSN	−1.48; −1.40
6;7	Ezrin	GI: 46249758	EZR	−1.60; **−**1.31
8	Synaptotagmin binding, cytoplasmic RNA interacting protein,isoform CRA_e	GI: 119569012	SYNCRIP	**−**1.33
9	Calreticulin precursor variant	GI: 62897681	CALR	**−**1.65
10	Heat shock protein 60	GI: 77702086	HSPD1	**−**1.47
11	Protein disulfide-isomerase A3 precursor	GI: 21361657	PDIA3	**−**1.62
12	T-complex protein1 isoform a	GI: 57863257	TCP1	**−**1.33
13	Nucleosome assembly protein 1-like 1	GI: 4758756	NAP1L1	**−**2.00
14; 15; 16	ACTB protein	GI: 15277503	ACTB	**−**1.44; −2.14; −1.87
17	Annexin VII isoform 1	GI: 4502111	ANXA7	−1.49
18	Alpha-enolase isoform 1	GI: 4503571	ENO1	−1.40
19	SET	GI: 145843637	SET1	−1.89
20	Nudix (nucleoside diphosphate linked moiety X)-type motif 5,isoform CRA_a*	GI: 119606726	NUDT5	−1.31
21	Serine/arginine-rich splicing factor 1 isoform 1	GI: 5902076	SFRS1	−1.37
22	Inorganic pyrophosphatase	GI: 11056044	PPA1	−1.36
23	Bisphosphoglycerate mutase	GI: 4502445	BPGM	−1.32
24	Phosphoglycerate mutase 1 (brain)	GI: 38566176	PGAM1	−1.51
25	Glutathione S-transferase	GI: 2204207	GSTP1	−1.42
26	Peroxiredoxin-1	GI: 4505591	PRDX1	−2.03
27; 28	Cellular nucleic acid-binding proteinisoform 1	GI: 187608726	CNBP1	−1.35; −1.32
29	Deoxyuridine 5'-triphosphate nucleotidohydrolase, mitochondrialisoform 2	GI: 4503423	DUT	−1.30
30	Hypothetical protein LOC51237*	GI: 117938314	MZB1	−1.57
31	Eukaryotic translation initiation factor 5A-1 isoform B	GI: 4503545	EIF5A	−2.10
32	Cofilin-1	GI: 5031635	CFL1	−1.35
33	60S acidic ribosomal protein P2	GI: 4506671	RPLP2	−2.08
34	Fatty acid-binding protein, epidermal	GI: 4557581	FABP5	−1.49
35	ISG15 ubiquitin-like modifier	GI: 14550514	ISG15	−1.48

* Not characterized as a protein.

Full list of candidates identified as being regulated by the BART miRNAs via 2D DIGE, along with changes in the protein levels observed in the presence of the miRNAs is shown.

**Table 2:** Candidate targets with predicted BART miRNA binding sites.

Gene	Predicted miRBARTs binding sites in the 3′UTR
CFL1	6-3p, 19-3p
CNBP1*	19-5p, 9, 3, 5
EIF5A	6-3p
GSNS1	11-3p (6 sites)
HSPA5	12, 1-3p
HSPA9	17-3p, 17-5p
NAP1L1*	12, 19-3p, 1-5p, 20-3p
PDIA3	14, 6-3p
PGAM1	11-3p
RPLP2	19-3p
SET1*	13, 6-5p
SFRS1*	1-5p
TCP1	12
CFL1	6-3p, 19-3p

* Gene with predicted miRBARTs, which are expressed at high levels in BJAB BARTs cells relative to that in BJAB Empty cells, binding sites in its 3′UTR.

A list of 13 candidates identified as putative targets which have predicted BART miRNA binding sites in the 3′ UTRs, along with the miRNAs predicted to target these transcripts are shown (*represent candidates selected for further validation).

### 2-dimensional Differential Gel Electrophoresis (2D DIGE) and Mass Spectrometry

2D DIGE and mass spectrometry protein identification was performed by Applied Biomics (Hayward, CA, USA). Briefly, protein extracts of BJAB BARTs and BJAB Empty cells were covalently linked to Cy3 and Cy5 CyDye respectively, mixed and run on first dimension isoelectric focusing, and second dimension SDS-PAGE in two technical replicates. Image scans were carried out following the SDS-PAGE using Typhoon TRIO (Amersham BioSciences), the scanned images analyzed by Image QuantTL software (GE-Healthcare), and subjected to in-gel analysis and cross-gel analysis using DeCyder software version 6.5 (GE-Healthcare). The ratio of the change of differentially expressed proteins was obtained from in-gel DeCyder analysis. To determine the ID of these differentially expressed proteins, selected spots were picked up by Ettan Spot Picker (GE-Healthcare) following the DeCyder software analysis and spot picking design. The selected protein spots were subjected to in-gel trypsin digestion, peptide extraction, desalting and followed by MALDI-TOF/TOF (Applied Biosystems) analysis to determine the protein identity.

**Table 3:** Immunoprecipitating RISCs from BJAB cells that express BART miRNAs constitutively.

		Fold Enrichment of miRNAs in RISCs*	
miRNA	Cell	Anti-GST	Anti-Ago2
miR-150	BJAB Empty	0.2	536
	BJAB BARTs	0.2	1052
BART10	BJAB Empty	1.3	2.1
	BJAB BARTs	2.1	869

* Fold enrichment of miRNAs was normalized to the amount of miRNAs in input.

Expression levels of cellular miR-150 and EBV’s miRBART10 were measured by stem-loop RT-PCR in BJAB BARTs and BJAB Empty RNAs immunoprecipitated with either anti-Ago2 or anti-GST antibody. Fold enrichment in each experiment was normalized to the Input sample. miR-150 is enriched in α-Ago2 immunoprecipitates in both BJAB BARTs and BJAB Empty cell lines, whereas BART10 is only enriched in immunoprecipitates of BJAB BARTs cells, as anticipated.

**Table 4:** Description of candidate mRNAs selected for further validation.

	Expression Level^a^				
Gene	BJAB Empty	BJAB BARTs	Fold Change	Predicted miRBARTs bindingsites in the 3′UTR^b^	Functions
CEBPA	1.69	62.24	36.73	3, 15	Transcription factor for cell growth and differentiation
TSPYL2	12.31	68.72	5.58	15, 5, 16, 19-3p, 7	Regulation of G1 checkpoint of cell cycle
IPO7	104.82	538.57	5.14	3, 16	Production of the proinflammatory cytokine IL-6;
					A member of β-karyopherinfamily that imports proteinsinto the nucleus
KISS1R	1.46	5.77	3.95	14	Receptor of KISS1 suppressor

a Expression level of mRNA was calculated using an algorithm as previously described (D. Vereide et al., manuscript submitted).

b Predicted EBV miRNAs targeted to cellular mRNA were analyzed by PITA algorithms (26).

The mRNAs enriched in the immunoprecipitates of RISCs from BJAB BART relative to BJAB Empty cells were evaluated based on the criteria described. Candidates selected for further validation are listed with their predicted binding sites for EBV miRNAs and their assigned functions.

**Table 5:** Putative targets enriched in RISCs of both BJAB BARTs and Jijoye cells.

	Fold Enrichment od mRNAs in RISCs*		
Gene	BJAB BARTs	Jijoye (15)	Predicted miRBARTs binding sites in the 3′UTR
IPO7	5.1	5.9	3. 16
PABPC4	3.0	3.4	9
GYG1	1.5	3.3	10
TOMM22	2.5	3.3	11-3p, 12, 16
ELP4	1.5	6.5	7, 12

* Fold enrichment of miRNAs was normalized to the amount of miRNAs in input.

Transcripts identified as enriched in RISC immunoprecipitations for both BJAB BARTs vs. BJAB Empty cells, and for Jijoye cells [15] are listed with their predicted mRNA binding sites and reported functions.

### Gene Expression Microarrays

mRNA microarrays were carried out as previously described (D. Vereide et al, manuscript submitted). Briefly, total RNA from BJAB BARTs and BJAB Empty cells was hybridized to whole genome human microarrays (Whole Human Genome Kit, 4×44 K features, 60-mer microarrays, Agilent, Foster City, CA) per manufacturer’s instructions. 150 ng of RNA was reverse-transcribed using a d(T)-T7 promoter primer into cDNA, and then transcribed into cRNA containing CTP labeled with either Cy3 or Cy5 by T7 RNA polymerase, using the Agilent Quick Amp Kit, Two color following the manufacturer’s protocol. Equal masses of Cy3- and Cy5- labeled cRNAs were co-hybridized to microarrays for the detection of mRNAs and scanned with an Agilent DNA Microarray Scanner. Microarrays were analyzed with EDGE3 software [23]. Statistical analyses were performed using Student’s T-test and revised false discovery rate (rFDR). The expression of mRNAs in BJAB BARTs and BJAB Empty cells was compared to the expression of mRNAs in a pooled sample of BJAB Empty cells in three independent experiments.

**Table 6:** Evaluating the utility of criteria used to reduce the number of candidate mRNAs.

Gene	Fold enrichment of mRNAsin RISCs^a^	Expression level ofencoded protein^b^	Predicted miRBARTs binding siteson the 3′UTR
ACTB	1.6	−1.4	None
ANXA7	1.5	−1.5	None
CALR	1.8	−1.7	None
CFL1	1.8	−1.4	6-3p, 19-3p
EIF5A	1.7	−2.1	6-3p
ENO1	2.3	−1.4	None
GSTP1	1.9	−1.4	None
PRDX1	2.0	2.0	None
RPLP2	1.8	−2.1	19-3p

a Fold enrichment of mRNAs in RISCs of BJAB BARTs cells relative to that of BJAB Empty cells were analyzed by deep sequencing.

b Expression level of proteins in BJAB BARTs cells relative to that in BJAB Empty cells were detected by 2D DIGE.

The genes identified as potential candidates for being regulated by BART miRNAs using both approaches are shown. Only 3 of these transcripts had predicted miRNA binding sites, however, these miRNAs were expressed inefficiently. None warranted selection for validation.

### RNA-Binding Protein Immunoprecipitation (RISC IP)

1×10^8^ cells were washed with PBS and lysed with 500 µl of PLB buffer (10 mM HEPES, pH 7.0, 100 mM KCl, 0.5% NP-40, 5 mM MgCl_2_, 200 U/ml RNase inhibitor (Ambion), 1 mM DTT, proteinase inhibitor cocktail) at 4°C for 30 min. Following centrifugation, the lysate was incubated with either anti-GST or anti-human Ago2 (11A9) antibodies (Provided by Dr. Gunter Meister [24]) conjugated dynabeads (Invitrogen) in 500 µl of NET-2 buffer (20 mM EDTA, pH 8.0, 1 mM DTT, and 200 U/ml RNase inhibitor in NT-2 buffer (150 mM Tris-HCl, pH 7.0, 100 mM Tris-HCl, pH 8.0, 750 mM NaCl, 0.25% NP-40, 5 mM MgCl_2_)) at 4°C for overnight. The protein-dynabead complexes were washed 6 times with NT-2 buffer, once with NTmS buffer (150 mM Tris-HCl, pH 7.0, 100 mM Tris-HCl, pH 8.0, 0.25% NP-40, 5 mM MgCl_2_) and resuspended with 1× Turbo DNase buffer and 2 U of Turbo DNase (Ambion) at 37°C for 30 min. The activity of DNase was eliminated with 15 mM of EDTA followed by addition of same volume of proteinase K buffer (2.4 mg/ml of proteinase K and 2% SDS in NT-2 buffer) at 55°C for 30 min. The RNA was extracted with acid-phenol/chloroform (pH 4.5) once, chloroform once and precipitated and resuspended in RNAse-free water.

### Illumina Deep-Sequencing

*A* cDNA library was prepared with Illumina mRNA-Seq sample preparation kit, according to the manufacturer’s protocol (Illumina), and examined by Illumina HiSeq2000 by the UWBC DNA Sequencing Facility. Sequence analysis was carried out using CLC Genomics Workbench 4.9 software as previously described (D. Vereide et al, manuscript submitted) to identify unique exon reads for each gene. The unique exon reads were used to determine expression values for each gene using the following formula: Expression value = (unique exon reads)×(10^9^)/(exon length)×(total unique exon reads).

### Stem-loop Real-time PCR

EBV BART miRNAs were measured as previously described [9]. The sequences of primers and probes are listed for each miRNA assayed (Table S2).

### Real-time PCR

1 ug of total RNA was reverse transcribed using Multiscribe (Applied Biosystems, Inc, High Capacity cDNA Reverse Transcription) according to manufacturer’s conditions using oligo d(T) at a final concentration of 5 uM. Reverse transcribed cDNAs were amplified and detected by qPCR under the following conditions: 1× Amplitaq Gold PCR Master Mix (Applied Biosystems), 0.5 µM each primer, 0.2 µM probe, 1× ROX reference dye (Invitrogen) and water to the final volume of 20 µl. PCR cycling conditions were 50°C for 2 minutes, 95°C for 10 minutes, and then 40 cycles of 95°C for 15 seconds, and 60°C for 60 seconds. Probes were labeled with 5′FAMRA and 3′TAMRA. The sequences of primers and probes are listed for each mRNA assayed (Table S3).

### Reporter Assays

Candidates found enriched in the RISCs by deep sequencing were tested by cloning their 3′UTRs downstream *of firefly* luciferase in the pmirGLO expression plasmid, which also contains an expression cassette for *renilla* luciferase as an internal control (Promega, Inc). 3′UTR-pmirGLO-derived constructs (100 ng) were transfected into the BJAB Empty and BJAB BARTs cell lines. 2.5×10^6^ cells were harvested 48 hours after electroporation, and the luciferase activity was measured using the Dual-Luciferase Reporter Assay System (Promega) following manufacturer’s protocol. *Firefly* activity relative to *renilla* was normalized to baseline luciferase activity achieved by transfecting pmirGLO construct alone (3′UTR-pmirGLO) and expressed relative to normalized activity in BJAB Empty cells, which was arbitrarily set to 100%. Each sample was measured twice per experiment, and each condition was repeated in three independent experiments.

### Immunoblotting

Cell lysates were separated on SDS-PAGE and transferred electrophoretically to PVDF membranes. The membranes were blocked in BLOTTO (5% nonfat milk, 0.05% Tween-20 in TBST buffer) and probed with anti-CEBPA (Acris Antibodies), anti-PUMA (Sigma-Aldrich), anti-CNBP1 (Sigma-Aldrich) or anti-Tubulin antibody (Sigma-Aldrich), followed by Horseradish peroxidase conjugated secondary antibody (KPL). The signals were detected using SuperSignal West Pico Chemiluminescent substrate (Thermo Scientific). Western blots were quantified using Image J software. Signals were determined to be in the linear range for their measurements when exposures for increasing times yielded directly proportional increases in signal intensities.

## Results

### Applying 2D DIGE to Identify Targets of BART miRNAs

Various proteomic approaches such as stable isotope labeling with amino acids in cell culture (SILAC) and two-dimensional gel electrophoresis (2D DIGE) followed by mass spectrometry for protein identification have been widely employed to characterize the effects of differential miRNA(s) expression on global protein abundance [17], [18], [25]. Because regulation by miRNAs does not necessarily lead to degradation of target transcripts, proteomic approaches potentially provide a comprehensive representation of effects of miRNA regulation. Indeed, works from the Rajewsky and the Bartel groups have shown that a large proportion of the regulation by cellular miRNAs is observed at the level of translation, underscoring the value of proteomic approaches [17], [18].

2D DIGE was selected for the comparison of techniques in this study in order to identify proteins that are differentially expressed in the presence of viral BART miRNAs. This method involves labeling two sets of protein samples using two different cyanine dyes, Cy3 and Cy5. The labeled samples can then be mixed allowing simultaneous separation of proteins by their isoelectric point as well as by their mass as a function of the SDS they bind. The samples can be visualized and quantified separately by using the excitation wavelength specific to each fluor. The analysis is reported to be more quantitative than the traditional 2D gel electrophoresis because of wide dynamic linear range and extreme sensitivity of the Cy3 and Cy5 fluors (reported to be 0.025 ng/spot) [25]. The most important virtue of this approach, however, is its ability to separate multiple pre- labeled samples on the same gel, which should significantly reduce the effects of the gel-to-gel variation observed with the traditional 2D gel electrophoresis [25].

The EBV-negative Burkitt’s lymphoma cell line, BJAB, which was either transduced with a retrovirus that expresses BART miRNAs constitutively (BJAB BARTs) or an empty virus vector (BJAB Empty) was used to identify cellular proteins whose expression levels are regulated by EBV’s miRNAs. This approach provided as isogenic cell lines as available currently, where changes in protein expression could be attributed to the effects of regulation by BART miRNAs alone, with no other viral genes being present. Expression of 26 BART miRNAs was extensively characterized in BJAB BARTs cells by stem-loop RT-PCR and compared to two EBV-positive Burkitt’s lymphoma cell lines, OkuI and SavI (Figure S1). These measurements were confirmed by doping known amounts of synthetic miRNA BART7 into initial extracts of cells and recovering more than 60% of the added miRNA in the final assays (Figure S1). Changes in the protein expression of 50% or less in the presence of BART miRNAs in BJAB BARTs cells in comparison to the BJAB Empty cells were sought, given that previous reports have found the effects of miRNAs on global protein expression are of this magnitude [17], [18].

2D DIGE and subsequent protein identification of differentially expressed spots was carried out by Applied Biomics, Inc. (Hayward, CA) (Table 1, Figure S2). This approach separated 2351 spots on the gels (Figure S2), which is consistent with the resolution achieved by other groups that have used this technique. Out of the total number of spots separated, 35 were decreased in intensity by 1.3-fold (30%) or more when compared to the BJAB Empty control which does not express EBV’s BART miRNAs (Table 1). The application of MALDI TOF/TOF mass spectrometry identified 28 unique and characterized proteins out of the 35 selected spots. This list of candidates was narrowed for further validation in as unbiased a manner as practical. Specifically, genes were selected for validation if their 3′UTRs were predicted to have sites complementary to the 3′ ends of BART miRNAs (Table 2). We used the Probability of Interaction by Target Accessibility (PITA) prediction algorithm to identify potential miRNA binding sites because this algorithm takes into account hybridization energy and binding site accessibility based on the secondary structure of the transcript, and does not rely on conservation of the miRNAs, a commonly used parameter not applicable to viral miRNAs [26]. We also evaluated the number of sites present in a given 3′UTR of a candidate mRNA, and whether the miRNAs predicted to target this gene are expressed efficiently or not (Figure S1). Four candidates were selected for further evaluation and examined by RT-PCR and/or immunoblotting (Table 2, Figure 1A, 1C).

### Identification of EBV BART miRNAs Targets by Immunoprecipitation of RISCs and Illumina Deep-sequencing

The 2D DIGE method resolves approximately a fifth to a quarter of the proteins present in a given cell [25], therefore 75–80% of the potential mRNA: miRNA interactions are missed by using this approach. Additionally, the list of identified proteins whose levels change will contain indirect targets of the miRNAs as well as spurious findings.

We used immunoprecipitation of RISCs and deep sequencing of the co-immunoprecipitated RNAs as a second approach to identify mRNAs potentially targeted by the BART miRNAs. A strength of this approach is that all mRNAs associated with EBV’s miRNAs in RISCs could be identified. A possible shortcoming of this approach is that it relies on a stable interaction among the Argonaute (Ago) proteins, the miRNAs, and the target mRNAs, in order to be efficiently immunoprecipitated. This difficulty can be overcome by cross-linking the complex by first exposing the cells to UV, though application of this technique has its own limitations such as low cross-linking efficiency and potential induction of the DNA damage response [12] [27].

To identify cellular genes targeted by EBV BART miRNAs, RISCs in BJAB BARTs and BJAB Empty cells were immunoprecipitated using anti-human Ago2 antibody or an irrelevant anti-GST antibody, which served as a negative control. The efficiency of immunoprecipitation was validated by stem-loop RT-PCR (Table 3), where cellular miR150, a miRNA highly expressed in B cells, and EBV BART10 miRNA in RISCs were precipitated by anti-Ago2 antibody but not by anti-GST antibody. Additionally, the levels of miR150 and BART10 miRNAs in RISCs were 2-fold and 400-fold higher in BJAB BARTs than BJAB Empty cells (Table 3). mRNAs which were enriched within the RISCs in the presence of the BART miRNAs were identified by sequencing using Illumina HiSeq2000 and analyzed using CLC Genomics Workbench 4.9 software. Expression levels of transcripts were determined as previously described (D. Vereide et al, manuscript submitted) to eliminate the detection from contaminated genomic DNA (Table 4, Table 5 and Table S1). 1405 mRNAs were identified as being enriched 1.5-fold or more in RISCs from cells expressing the BART miRNAs (Table S1). Importin 7 (IPO7) and Mitochondrial import receptor subunit TOM22 homolog (TOMM22), two genes which are involved in cellular transport that were recently reported as targets of BART miRNAs in Jijoye cells, and validated by RT-PCR and reporter assays [15], were both found enriched in RISCs in BJAB BARTs cells by 5.1-fold and 2.5-fold respectively in comparison to BJAB Empty cells (Table 4, Table 5). Along with IPO7 and TOMM22, we detected enrichment for another 3 genes (PABPC4, GYG1 and ELP4) in BJAB BARTs RISCs that were identified as candidates for regulation by the BART miRNAs in Jijoye and B95-8 cell lines by the Hass group (Table 5). Interestingly, we found no enrichment of PUMA (p53-mediated modulator of apoptosis) transcripts in the presence of BART miRNAs, which is a pro-apoptotic gene reported to be regulated by BART5 in epithelial cells [13]. Table 4 describes transcripts and their reported functions, which were enriched in RISCs in the presence of the BART miRNAs and chosen for further validation. These candidates were selected due to their high level of enrichment in RISCs in the presence of BART miRNAs, as well as the stability of predicted miRNA binding sites in their 3′UTRs.

### Measuring Levels of Candidate Targets with Microarrays

The levels of mRNAs that are candidates for being regulated by BART miRNAs may be affected by these miRNAs. If these levels were affected by other means, then differences in them could confuse the identification of the mRNAs as being bona fide targets. Decreases in the levels of a particular candidate mRNA could lead to a decrease in the level of the protein it encodes or to its decrease in a RISC, for example. We measured the levels of all identified candidate mRNAs with microarrays and found no differences in the level of candidates found with 2D DIGE, but found decreases in the levels of 16 mRNAs enriched at low levels (∼2.75-fold or less) in immunoprecipitates of RISC from BJAB BARTs cells (Table S4). The decrease of the levels of these 16 candidates may result from the BART miRNAs that could target them or from some other means. If the latter were correct then the diminution in levels of bona fide target mRNAs could limit their recognition as such because of decreased levels in RISCs.

### Validation of EBV miRNA Target Candidates by RT-PCR, Dual-Luciferase Assays, and Immunoblotting

Levels of the candidate transcripts (IPO7, PUMA, CNBP1, NAP1L1, SET1, SFRS1) were measured by RT-PCR in the Ago-immunoprecipitated RNA samples from both BJAB BARTs and BJAB Empty cells, in order to assess the degree of enrichment of these transcripts in the RISCs of cells expressing BART miRNAs (Figure 1A). Individual transcript levels were normalized to the levels of a housekeeping gene, Hypoxanthine Phosphoribosyl transferase 1 (HPRT1). The mRNA encoding IPO7 was enriched ∼13-fold (p value <0.005, Student’s t-test) in cells expressing BART miRNAs compared to the BART Empty cells, however there was no detectable enrichment of the mRNAs encoding the targets identified via 2D DIGE (CNBP1, SET, SFRS1, and NAP1L1), indicating that the variation in detection by 2D DIGE with two technical replicates is not supported by data from RISC immunoprecipitations (Figure 1A).

In order to validate candidate targets identified by either approach, full length 3′UTRs of candidate genes were cloned downstream of *firefly* luciferase ORF into pGL3-Control (Promega) or a dual-luciferase vector pmirGLO (Promega) respectively. Candidates identified from RISC immunoprecipitations were selected on the basis of their enrichment and predicted binding sites for BART miRNAs (Table 4). These reporter constructs were transfected into BJAB BARTs and BJAB Empty cells, with the *firefly* expression normalized to levels of *renilla* activity. It is important to note that these constructs were assayed in cells that express physiological levels of miRNAs (BJAB BARTs) (Figure S1). While no effect on luciferase activity was detectable for reporter constructs of SET1 and PUMA (data not shown), CEBPA, TSPYL2, and KISS1R, we observed a reproducible ∼33% decrease of luciferase expression in BJAB BARTs cells when transfected with IPO7-pmirGLO reporter construct (Figure 1B).

We tested the direct effects of BART miRNAs on the protein levels of candidate targets by immunoblotting where appropriate antibodies were available using three biological replicates. Levels of CEBPA, PUMA, CNBP1, and IPO7 were measured in the cell extracts of BJAB Empty versus BJAB BARTs cell lines (Figure 1C). Expression levels of these proteins were quantified using Image J software and normalized to those of alpha-tubulin. While the expression of CEBPA, PUMA, and CNBP1 remains unaltered in the presence of BART miRNAs, we detected a 33% decrease in the levels of IPO7 in BJAB BARTs cells in comparison to the control cell line. The failure to observe a decrease in the levels of CNBP1 in the presence of BART miRNAs by immunoblotting likely reflects a decreased precision in the results of 2D DIGE when carried out with two technical replicates. The results of the different validation experiments are summarized in Figure 1D, with IPO7 confirmed as a bona fide target of BART miRNAs in the three different validation experiments.

### Analysis of Candidates Obtained by 2D DIGE and RISC IPs by Bioinformatics

We looked for any insights in comparing our two approaches we could glean from analyzing the candidates they uncovered using the program PITA. 3′UTRs of 35 candidates identified by 2D DIGE and of 135 candidates identified by RISC IPs, which were enriched at least at levels comparable to TOMM22 were screened for predicted binding sites of BART miRNAs. Approximately 46% of candidates identified by 2D DIGE were predicted to have binding sites for BART miRNAs (Table 2), compared to approximately 40% among the candidates identified by RISC IPs (data not shown). Neither approach shows an advantage in identifying candidates predicted by PITA to be bound by BART miRNAs.

## Discussion

Studies of levels of proteins and of mRNAs in RISCs have been used to implicate cellular miRNAs as regulators of the levels of specific proteins. These studies have been facilitated by the conservation of miRNAs and of their targets during evolution. For example the let-7 miRNA is conserved from *C. elegans* to people [28]. miRNAs encoded by viruses such as EBV have no equivalent conservation to aid in the identification of the mRNAs they target. We have tested proteomic analysis and RISC immunoprecipitation coupled with deep sequencing to gauge which approach is more practical for identifying targets of EBV’s BART miRNAs.

We used an EBV-negative Burkitt’s lymphoma cell line, BJAB, which was transduced either with a retroviral vector encoding viral BART miRNAs or an empty vector in this comparison. We first measured the expression level of all the BART miRNAs by stem-loop RT-PCR in these BJAB cells to ensure that the expression of the miRNAs was comparable to those observed in EBV-positive BLs. Most EBV miRNAs are expressed at low levels relative to many cellular miRNAs (Figure S1) [9]. Many studies achieved differential expression of a miRNA(s) of interest often by transient transfection of synthetic analogs, leading to the expression of the miRNAs at higher than physiological levels.

These cells were analyzed with 2D DIGE followed by mass spectrometry to identify proteins that differed by 1.3-fold or more in their levels between BJAB cells expressing the BARTs and sister cells that did not express them. Of 35 signals that were decreased in the presence of the BART miRNAs, 28 were identified by mass spectrometry as unique proteins, 13 of which had predicted binding sites for BART miRNAs. However, the levels of their mRNAs were the same in each cell type, none was found to be enriched in RISCs from cells expressing the BARTs, and none was found to be regulated by the BART miRNAs in luciferase assays. These cells were analyzed in parallel by immunoprecipitating RISCs from them and identifying mRNAs in the complexes by deep sequencing. 1405 mRNas were enriched by 1.5-fold or more in the RISCs from BJAB cells expressing the BART miRNAs. Immunoprecipitating RISCs followed by deep sequencing of enriched mRNAs was more informative, identifying multiple targets we and others have confirmed [15], (D. Vereide et al, manuscript submitted). Since mRNAs identified through this process were precipitated as part of the RISCs, these candidates may be enriched for direct targets of miRNAs though indirect targets will be present, too. Using this approach IPO7, a gene involved in cellular transport, was enriched ∼5-fold in RISCs in the presence of BART miRNAs, and was validated as a target of EBV’s miRNAs by both reporter assays and direct measurement of IPO7 protein in the two cell-types. Along with IPO7, four additional genes also reported as candidate targets of BART miRNAs in Jijoye cells by J. Haas et al were found to be enriched (Table 5) [15].

We assessed the efficiency of the RISC IPs we used to ones in which RNAs are cross-linked to protein prior to immunoprecipitation by comparing the number of unique reads obtained for the cDNAs derived from the immunoprecipitations for each approach. The RISC IPs without prior cross-linking achieved a comparable number of unique reads to those obtained by two recent reports which have used either UV or PAR crosslinking (16 million reads in this study, 9–30 million in these other studies [12], [16]). These latter methods used gel purification of the immunoprecipitated complexes prior to isolating RNA which would decrease their efficiency and compensated for this loss by beginning with approximately 10-fold more cells than did we. Along with the Haas group, we have also used the PITA algorithm to identify potential direct targets of EBV’s miRNAs, which is reported to be more conservative than the algorithm applied in the studies using cross-linking [12], [16], [29]. While these differences preclude a direct comparison, the comparable number of unique reads found by these different approaches indicates that they do not differ grossly in their efficiencies of isolating mRNAs in RISCs. This contention is supported also by the work of Haas and colleagues [15] who identified 30–50% of the number of candidates found in the studies using cross-linking [12], [16].

The methods described here along with other techniques commonly applied in attempts to identify targets of miRNAs are surveys of either individual or global changes mediated by the miRNAs. These approaches yield large number of candidates, which create a need for discerning criteria that would practically reduce the list of candidates selected for further validation. Candidates obtained by both 2D DIGE and RISC IPs in this study were evaluated for the presence of predicted miRNA binding sites, as well as by the number of different viral miRNAs predicted to bind a target 3′ UTR, and their respective level of expression (Table 2, Table 4, Figure S1) [26]. Comparison of candidates identified by RISC IPs and 2D DIGE in BJAB BARTs cells to the previously reported findings allowed us to evaluate the usefulness of these criteria in reducing the number of candidates selected for validation [15]. No candidates identified initially by both approaches, for example, warranted validation based on these criteria (Table 6).The only criterion we find useful is the presence of a binding site of a miRNA in a candidate’s 3′ UTR which is predicted to be particularly stable [26]. This feature alone, however, does not define a candidate as being a target for that miRNA.

Neither the number of different miRNAs predicted to bind to the target 3′UTR nor the expression levels of these miRNAs were found to be compelling indicators of a candidate’s being regulated by these miRNAs, too. For example, we and other groups have identified and validated IPO7 as a target of BART miRNAs, which is predicted to be regulated by only two viral miRNAs, BARTs 3 and 16 [15], (D. Vereide et al, manuscript submitted). While both miRNAs are expressed similarly (Figure S1), only the BART3 miRNA binding site in IPO7’s 3′UTR is regulated by the viral miRNAs in reporter assays [15]. These findings indicate that it is essential to develop functional assays to identify the mRNAs targeted by EBV’s miRNAs in which the viral miRNAs are expressed at physiological levels.

## Supporting Information

Figure S1 **Measuring levels of BART miRNAs using stem-loop RT-PCR.** A. 26 BART miRNAs were measured in BJAB cells infected with a retroviral vector encoding both clusters of BART miRNAs by stem-loop RT-PCR. Known quantities of synthetic miRNAs were used to generate standards and used for quantification as previously described [9]. B. Known amounts of synthetic BART7 (1000 copies/10 pg) were added to cell extracts of BJAB and OkuI cells, which express no BART7 and ∼248 copies/10 pg of BART7 respectively, to measure the efficiency of recovery of this miRNA. At least 60% of the expected quantity of BART7 miRNA was detected by stem-loop RT-PCR in both OkuI and BJAB, indicating that our measurements of EBV miRNAs are accurate likely within a factor of 2.
(XLSX)

Figure S2 **Image scans of the 2D gels along with the overlay image are shown.** Protein extracts from BJAB Empty and BJAB BARTs cells were labeled with Cy5 and Cy3 respectively, and analyzed by 2D DIGE. The purple circles represent spots which were selected for identification by mass spectrometry.
(TIF)

Table S1 **Full list of 1405 candidates identified as being regulated by the BART miRNAs by virtue of their being enriched in RISCs following immunoprecipitation by anti-Ago2.**
(XLSX)

Table S2 **Sequences of stem-loop RT-PCR primers and probes used to measure BART miRNAs.** Additional sequences used in this study were previously reported [9].
(XLSX)

Table S3 **Sequences of RT-PCR primers and probes used to measure expression levels of candidate genes obtained via 2D DIGE and RISC IPs.**
(XLSX)

Table S4 **A list of candidate genes which decreased in expression in the presence of BART miRNAs as detected by microarrays, and which have also been shown to be enriched in RISCs of BJAB BARTs cells.**
(XLSX)
