# Supplementary figures and images for: Comparing Proteomics and RISC Immunoprecipitations to Identify Targets of Epstein-Barr Viral miRNAs
Source: PLoS One. 2012 Oct 16;7(10):e47409. doi: 10.1371/journal.pone.0047409 (PMC3472983; doi:10.1371/journal.pone.0047409)

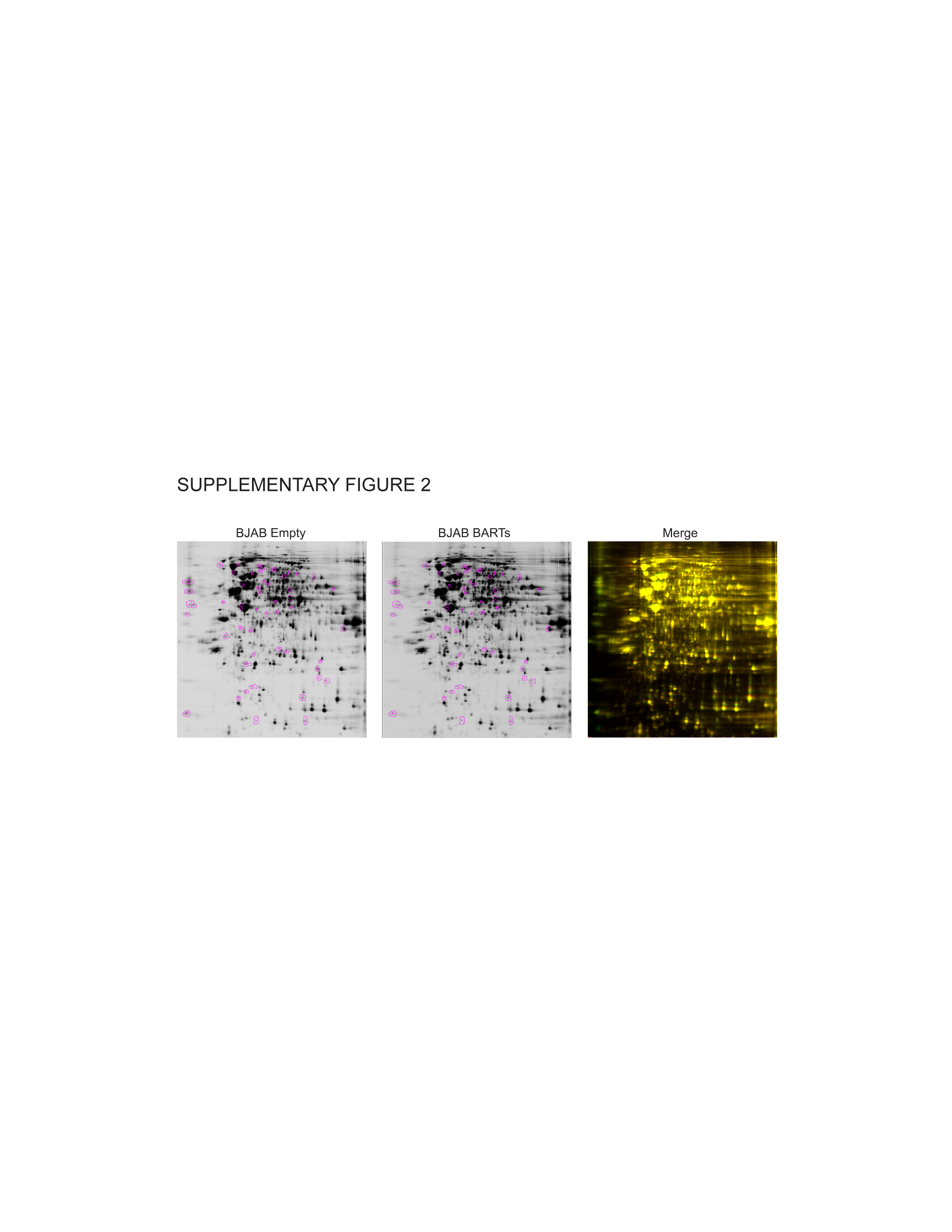

Supplement: Figure S2 — Image scans of the 2D gels along with the overlay image are shown. Protein extracts from BJAB Empty and BJAB BARTs cells were labeled with Cy5 and Cy3 respectively, and analyzed by 2D DIGE. The purple circles represent spots which were selected for identification by mass spectrometry. (TIF) [file pone.0047409.s002.tif]
